# ISRIB facilitates post-spinal cord injury recovery through attenuation of neuronal apoptosis and modulation of neuroinflammation

**DOI:** 10.1016/j.jot.2025.01.003

**Published:** 2025-03-07

**Authors:** Qingyang Li, Chi Zhang, Enlin Qi, Mingxin Wu, Haijian Sun, Tao Zhang, Yunpeng Jiang, Hao Li, Ruizhi Jiang, Chuang Li, Hua Zhao, Hengxing Zhou, Shiqing Feng

**Affiliations:** a Department of Orthopaedics, Qilu Hospital of Shandong University, Shandong University Centre for Orthopaedics, Advanced Medical Research Institute, Cheeloo College of Medicine, Shandong University, Jinan, 250012, PR China; b Department of Orthopaedics, The Second Hospital of Shandong University, Cheeloo College of Medicine, Shandong University, Jinan, Shandong, 250033, PR China; c Center for Reproductive Medicine, Shandong University, Jinan, Shandong, 250012, PR China; d Department of Orthopaedics, Tianjin Medical University General Hospital, International Science and Technology Cooperation Base of Spinal Cord Injury, Tianjin Key Laboratory of Spine and Spinal Cord, Tianjin, 300052, PR China

**Keywords:** Spinal cord injury, ISRIB, Neuronal apoptosis, Neuroinflammation, RNA sequencing

## Abstract

**Background:**

Neuronal apoptosis and inflammation are two critical factors that impede functional recovery post spinal cord injury (SCI). Previous studies have demonstrated the inhibitory effects of integrated stress response inhibitor (ISRIB) on neuroinflammation in brain injury. However, whether ISRIB can regulate neuron death and neuroinflammation in the context of SCI remains elusive.

**Methods:**

We employed an oxygen-glucose deprivation/reperfusion (OGD/R) model to simulate spinal cord ischemia-reperfusion injury and utilized lipopolysaccharide (LPS) to activate microglia. We assessed cell viability and death to demonstrate the neuroprotective effect of ISRIB against neuron death, while evaluating cytokine levels and the expression of Arg1 and iNOS to elucidate the regulatory role of ISRIB in neuroinflammation. Bulk RNA-seq analysis was employed to investigate the global transcriptional changes in neurons and microglia induced by ISRIB treatment. Additionally, we validated the promoting effects of ISRIB on motor and sensory recovery in a mouse model of SCI.

**Results:**

We observed that ISRIB exerted a suppressive effect on neuron death and neuroinflammation. RNA-seq data revealed that the ISRIB exhibited regulation of neuron apoptosis through the P53 signaling pathway, as well as modulation of neuroinflammation by the JAK2/STAT3 signaling pathway. Western blotting and immunofluorescence analyses demonstrated that ISRIB reduced P53 expression in neuronal nuclei and inhibited the phosphorylation of JAK2 and STAT3 in microglia. In addition, we validated the capacity of ISRIB to promote locomotor function recovery in a mouse model of SCI.

**Conclusion:**

Our study confirmed the ability of ISRIB to regulate neuron apoptosis and neuroinflammation in SCI via the P53 signaling pathway and the JAK2/STAT3 signaling pathway, respectively. Treatment with ISRIB in mice with SCI promoted the recovery of neural function. This research provides new evidence and options for therapeutic strategies of SCI.

**The translational potential of this article:**

Our study provides experimental evidence to support the application of ISRIB in the repair of spinal cord injury.

## Graphical abstract

**Figure:**
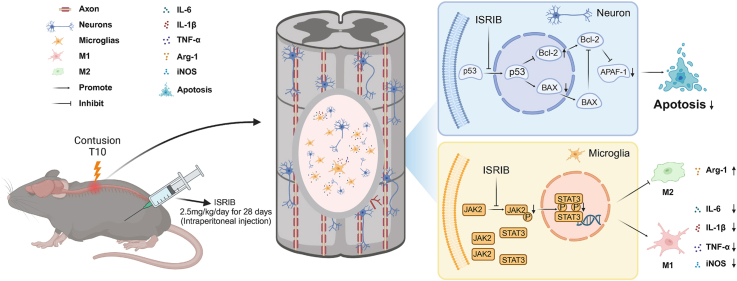

## 1 Introduction

Spinal cord injury (SCI) is a devastating neurological disorder [1], which leads to sensory and locomotor function impaired. According to the latest epidemiological research, there are about 0.9 million new cases of SCI in the world, with a total number of patients reaching 20.6 million [2]. Patients who suffered acute traumatic SCI cost an average of 71,300 CNY/11,500 USD [3]. Such substantial expenses impose a heavy burden on both patients' families and society.

The pathophysiology of SCI is highly intricate. In general, it can be categorized into primary injury and secondary injury. Most of primary injuries result from direct external forces [4], which caused spinal cord ischemia [5], edema [6], axon disruption [7] and disruption of blood–spinal cord barrier [8]. Ischemia-reperfusion is a prevalent pathologic process in SCI [9,10], and it plays a crucial role in secondary injury. Neural cells experience inadequate energy supply due to ischemia. While the perfusion of the spinal cord leads to an overproduction of reactive oxygen species (ROS). Excessive ROS production damages DNA and organelles, ultimately triggering neural cell death [11]. The apoptosis of neurons during spinal cord ischemia-reperfusion is a decisive factor in the recovery of neurological function after SCI. Therefore, exploring methods to inhibit neuronal apoptosis has always been a major concern in the field of SCI research [[12], [13], [14]].

Inflammation is closely associated with the progression the SCI, the intensity of immune inflammatory reaction directly influences the extent of injury. Resident microglia respond rapidly and are activated into pro-inflammatory cells in a short time after SCI [15]. Microglia begin to for IL-1β, IL-6 and TNF-α within hours [16]. These three extensively studied pro-inflammatory cytokines that attract microglia and macrophages to infiltrate the injury site, thereby initiating inflammatory cascades. Microglia have two polarized states: the M1 phenotype, which exacerbate neuroinflammation, and M2 phenotype, which promote inflammation resolution and tissue repair [17]. A large number of microglia polarize to the M1 subtype following SCI, which further exacerbates local inflammatory responses and impedes neural repair. Therefore, it is reasonable to inhibit overactive inflammatory response and maintain the balance of M1 phenotype and M2 phenotype.

Previous studies reported the pharmacological properties of integrated stress response inhibitor (ISRIB), a small molecule compound that acts by inhibiting the integrated stress response. Initially, ISRIB was found to effectively suppress the phosphorylation of eukaryotic translation initiation factor 2α (EIF2α) [18]. EIF2α is a pivotal molecule in the integrated stress response, and phosphorylation of EIF2α has been shown to impair memory. So, ISRIB was used to enhance memory in rodent [19]. Thereafter, ISRIB was used in other neurological diseases, such as neurodegeneration [20] and cognitive deficits [21]. Although previous studies have indicated that ISRIB alleviated neuronal apoptosis by regulating the integrated stress response [22,23], alternative potential mechanisms may also be involved in the regulation of neuronal death. Furthermore, it was unclear whether ISRIB exerted regulatory effects on neuroinflammation following SCI, and what the underlying regulatory mechanisms might be. In light of these considerations, it was essential to investigate the regulatory mechanisms of ISRIB on neuronal apoptosis and neuroinflammation after SCI.

In this study, we aimed to explore the role of ISRIB in promoting neural functional recovery following SCI and to elucidate its underlying molecular mechanisms. Our findings demonstrated that ISRIB could effectively alleviate neuronal apoptosis induced by OGD/R and inhibit the activation of M1-type microglia induced by LPS. Furthermore, we elucidated that ISRIB exerted its effects on promoting neuronal survival and reducing inflammatory responses through two different signaling pathways. The findings revealed that ISRIB suppressed apoptosis via the P53 signaling pathway. Furthermore, ISRIB was observed to modulate neuroinflammation following SCI through the JAK2/STAT3 signaling pathway, which was previously unreported. In vivo experiments further revealed that intraperitoneal injection of ISRIB promoted motor and sensory function recovery in mice with SCI. In conclusion, our study provided biological evidence supporting the potential of ISRIB as a therapeutic option for SCI.

## 2 Materials and methods

### 2.1 Animals

The C57BL/6 mice (6–8 weeks, female) were purchased from Beijing Vital River Laboratory Animal Technology. Mice were kept in an environment with 12 h light/12 h dark cycle and controlled temperature. Food and water were free accessed to animals. All experiments were approved by Medical Ethics Committee of Qilu Hospital of Shandong University (NO. DWLL-20210061) and performed according to the national guidelines of use and care for laboratory animals.

### 2.2 SCI model

The C57BL/6 mice were anesthetized at 4 % isoflurane (RWD, 510-22) and maintained at 2 % isoflurane. A laminectomy was used for exposure of the T10 spinal cord, followed by contusion injury at this level using an impactor (RWD, 68099Ⅱ-S-M). Parameters of impactor: diameter, 1.0 mm; velocity, 1.5 m/s; duration, 0.5 s; depth, 0.5 mm. After the operation, the mice were placed on heating pad until mice revived. Bladder massage was performed to facilitate urination twice daily following the injury.

### 2.3 Drug administration

ISRIB (MedChemExpress, HY-12495) solution and vehicle were made up according to previous study [24]. ISRIB used for in vitro studies was dissolved in DMSO to a concentration of 10 mM, and then diluted to 200 nM with cell culture medium. For in vivo studies, ISRIB was dissolved in a 1:1 mixture of DMSO and PEG400, and the same mixture of DMSO and PEG400 was used as the Vehicle. Animals of SCI received intraperitoneal injection with ISRIB at 2.5 mg/kg or vehicle daily for a duration of 28 days.

### 2.4 Cell culture

HT22 and BV2 were purchased from SHANGHAI WHELAB BIOSCIENCE LIMITED. HT22 and BV2 were cultured in high-glucose Dulbecco's modified Eagle's medium (DMEM, BasalMedia, L110KJ) supplement with 10 % fetal bovine serum (FBS, Cellmax, SA101.02), 1 % penicillin/streptomycin (Solarbio, P1400) at 37 °C, 5 % CO_2_.

### 2.5 Isolation of primary cortical neurons from mice

Primary cortical neurons were isolated from fetal (embryonic day 17 [E17]) mice as previously described [25]. After the pregnant mice were euthanized, the uterus was removed and placed in pre-chilled PBS. And then, cerebral cortex was separated. The cortex was cut into small pieces, papain (Worthington, LS003119) and DNase Ⅰ (Sigma, D5025) were used to dissociate cells. High-glucose DMEM supplement with 10 % FBS was used for neuron culture. After 4 h, medium was replaced with neurobasal medium (Gibco, 21103049) supplement with B27 (Gibco, 17504044) and L-glutamine (Gibco, 25030081).

### 2.6 Oxygen-glucose deprivation/reoxygenation (OGD/R) model

The medium of HT22 cells was replaced with glucose-free DMEM (BasalMedia, L160KJ), HT22 cells were incubated at GENbag anaerobic incubation system (bioMérieux SA, 45534) for 6 h at 37 °C, and replaced with high-glucose DMEM, cells were cultured in incubator (37 °C, 5 % CO2) for 18 h.

On the sixth day after the isolation of the primary neurons, OGD/R model was performed. The neurons were washed for 3 times with PBS (Solarbio, P1020) and glucose-free DMEM was added, then neurons were incubated in incubator at 37 °C, 1 % O_2_ and 5 % CO_2_ for 1 h. Neurons were washed for 3 times with PBS and replaced with normal medium for 24 h in the incubator (37 °C, 5 % CO2).

### 2.7 Cell counting kit-8(CCK8)

CCK8 (Proteintech, PF00004) was incubate with cells for 1 h at 37 °C and absorbance was detected at the wavelength of 450 nm by microplate reader (TECAN, INFINITE 200 PRO).

### 2.8 Calcein-AM/PI staining assay

Calcein-AM/PI Assay Kit (Beyotime, C2015S) was used to detect live and dead cells. Cells were washed once, and HT22 cells incubated with Calcein/PI detection solution at 37 °C for 30 min, photos were captured by inverted fluorescence microscope (ZEISS, Axio Vert.A1).

### 2.9 Reactive oxygen species (ROS) detection

HT22 cells were incubated with DCFH-DA (Beyotime, S0033S) at 37 °C for 20 min, and washed by DMEM for 3 times, and observed with inverted fluorescence microscope (ZEISS, Axio Vert.A1).

### 2.10 RNA extraction and RT-qPCR

RNA was extracted by Total RNA Extraction Kit (Solarbio, R1200), Revert Aid First Strand cDNA Synthesis Kit (Thermo Scientific, K1622) was used to RNA reverse transcription. SYBR®Green (Accurate Biology, AG11704) was used to perform qPCR. β-Actin was control. Data analysis was used 2^^-△△Ct^. The primers used for qRT-PCR was shown in Table 1.

**Table 1:** The primers of genes.

Gene	Forward (5′-3′)	Reverse (5′-3′)
*IL-1β*	GCAACTGTTCCTGAACTCAACT	ATCTTTTGGGGTCCGTCAACT
*IL-6*	TAGTCCTTCCTACCCCAATTTCC	TTGGTCCTTAGCCACTCCTTC
*TNF-α*	CCCTCACACTCAGATCATCTTCT	GCTACGACGTGGGCTACAG
*IL-4*	GGTCTCAACCCCCAGCTAGT	GCCGATGATCTCTCTCAAGTGAT
*IL-10*	GCTCTTACTGACTGGCATGAG	CGCAGCTCTAGGAGCATGTG
*Arg1*	CTCCAAGCCAAAGTCCTTAGAG	AGGAGCTGTCATTAGGGACATC
*iNOS*	GTTCTCAGCCCAACAATACAAGA	GTGGACGGGTCGATGTCAC
*β-actin*	GGCTGTATTCCCCTCCATCG	CCAGTTGGTAACAATGCCATGT

### 2.11 Library preparation and sequencing

To construct mRNA libraries and perform sequencing, RNA samples were prepared using the TruSeq RNA Sample Preparation Kit. Poly-A-containing mRNA molecules were isolated using magnetic beads coated with poly-T oligonucleotides. First strand cDNA was reverse transcribed using RNA fragments, followed by the synthesis of second strand cDNA. The resulting cDNA fragments were subjected to purification, end blunting, A-tailing, and adaptor ligation. DNA fragments with adapter molecules on both ends were enriched and the library's DNA content was amplified by PCR. To avoid introducing bias into the library representation, the cycles of PCR was minimized. Quality assessment of the libraries was performed using an Agilent 2100 bioanalyzer, while quantification was carried out using Qubit and qPCR. Finally, HiSeq 2500 platform was used for sequencing.

### 2.12 RNA-seq data analysis

To transform the raw data of fastq format into clean reads, the data was subjected to initial processing using custom Perl scripts. The clean data was used for subsequent downstream analyses. The paired-end clean reads were processed by Hisat2 (v2.0.5) to aligned to the reference genome. To quantify the number of reads mapped to each gene, FeatureCounts, a component of Subread (v2.0.4), was utilized. The FPKM value was determined by gene length and the count of reads mapped to gene. Subsequently, differential expression analysis was carried out by the DESeq2 R package (v1.4.5). Benjamini-Hochberg method was used for P-values adjustment. For gene set enrichment analysis (GSEA), gene sets were obtained from the MSigDB Database (https://www.gsea-msigdb.org/gsea/msigdb). Prior to analysis, all genes were ranked by logFC values. GSEA analysis was performed using the clusterProfiler package (v4.8.1). Additionally, to identify pathway changes, GSVA R package (v1.48.1) was used to conduct gene set variation analysis (GSVA).

### 2.13 Western blotting (WB)

RIPA lysis buffer (Beyotime, P0013B) containing 1× phosphatase inhibitor cocktail (ROCHE, P5726) was incubated with cells on ice. The supernatant was obtained after centrifugation at 12,000 rpm for 20 min. Then, 5× loading buffer was mixed with supernatant, and protein was incubated at 100 °C for 5 min. All samples were separated using SDS-PAGE, and transferred into PVDF Membrane. The membranes were blocked by 5 % milk. And then, membranes were incubated with β-actin (1:1000, CST, 4970), BAX (1:1000, Proteintech, 60267-1-Ig), cleaved Caspase3 (1:1000, CST, 9661), P53 (1:5000, Proteintech, 21891-1-AP), Histone-H3 (1:2000, Proteintech, 17168-1-AP), JAK2 (1:1000, CST, 3230), p-JAK2 (1:1000, CST, 4406), STAT3 (1:2000, Proteintech, 10253-2-AP), p-STAT3 (1:2000, CST, 9145) at 4 °C overnight, and then incubated with second antibodies at room temperature for 1 h. The greyscale of the proteins was analyzed using the ImageJ software (version 1.53e). The data for each group were normalized to the mean value of three replicates from the Control group.

### 2.14 Mitochondrial depolarization assay

Mitochondrial depolarization was assessed by JC-1 kit (Beyotime, C2006). In brief, cells were incubated with JC-1 solution in the incubator for 20 min. Medium was added after cells were washed twice. Finally, the photos were captured using fluorescence microscope.

### 2.15 TUNEL staining

Cells were fixed with 4 % Paraformaldehyde (PFA) (Solarbio, P1110) and permeabilized with 0.5 % Triton X-100. And then, cells were reacted with TUNEL detection solution (Beyotime, C1089) for 1 h at 37 °C, 4’,6-diamidino-2-phenylindole (DAPI) (Beyotime, C1002) was used to stain nucleus. The TUNEL staining positive cells were detected by fluorescence microscope.

### 2.16 Immunofluorescence

The cells were fixed with 4 % PFA (Solarbio, P1110) and blocked with a solution containing 10 % goat serum (Solarbio, SL038) and 0.5 % Triton X-100 (Solarbio, T8200) for 1 h at room temperature. Following this, the cells were incubated overnight at 4 °C with Arg1 (1:500, Proteintech, 16001-1-AP), iNOS(1:500, Proteintech, 22226-1-AP), P53 (1:500, Proteintech, 21891-1-AP). And appropriate secondary antibodies were used to incubate cells at room temperature for 1 h. Finally, DAPI was used for nuclear staining for 10 min. The photos of Arg1 and iNOS were captured by fluorescence microscope, photos of P53 were captured by laser scanning confocal microscope (Olympus, SpinSR10). The fluorescence intensity was analyzed using ImageJ 1.53e software.

### 2.17 Immunofluorescence staining of tissue

At the 42 days post spinal cord injury (dpi), mice were anesthetized and perfused by PBS and 4 % PFA, Tissues were fixed in 4 % PFA solution for 1 day, and dehydrated with 30 % sucrose solution for 2 days. Spinal cord tissue was embedded with OCT (SAKURA, 4583) and was cut for 9 μm thickness. The tissue sections were permeabilized and blocked, followed by the incubation with IL-1β (1:400, Proteintech, 26048-1-AP), NeuN (1:400, Abcam, ab104224), iNOS (1:400, Abcam, ab210823), IBA1 (1:400, Abcam, ab178846), cleaved Caspase3 (1:400, CST, 9661), p-STAT3 (1:400, CST, 9145), P53 (1:500, Proteintech, 21891-1-AP) and appropriate secondary antibodies. DAPI was used for nuclear staining for 10 min The photos were captured by fluorescence microscope. The fluorescence intensity was analyzed using ImageJ 1.53e software. To ensure comparability, the data for each group were normalized using the mean value of three replicates from the Sham group.

### 2.18 Hematoxylin-eosin (HE) staining

The mice were reperfused by PBS and 4 % PFA, spinal cord tissues and bladders were fixed in 4 % PFA for 1day, spinal cord tissues and bladders were soaked by 30 % sucrose solution for two days. OCT was used to embed tissues, and tissue was cut for 9 μm thickness by freezing microtome (Leica, CM3050S). The steps of Hematoxylin-Eosin (HE) staining according to a method before [26]. The photos were captured by microscope (Olympus, BX63).

### 2.19 Basso mouse scale (BMS) scoring analysis

BMS score was used to assess locomotor function recovery of hindlimb after SCI in mice. Briefly, the mice were assessed by two trained experimenters for a duration of 4 min in an empty room. All animals were scored from 0 to 9.

### 2.20 Catwalk gait analysis

At 42 dpi, gaits and motor function were evaluated by Catwalk XT system (Noldus, version 10.6). During the experimental procedure, the animals were positioned within a walkway and filmed from a ventral perspective. Parameters of behavior, such as the regularity index and base of support, were evaluated. Regularity index was used to evaluate forelimb–hindlimb coordination according to normal step sequence patterns (NSSPs), base of support was used to evaluate average width between the hind paws.

### 2.21 Electrophysiological tests

Electrophysiology assay was performed at 42 dpi by using BL-420 biological function experimental system (TECHMAN). Mice were anesthetized by Isoflurane (RWD, R510-22-10). For stimulating and recording, one side of the sciatic nerve was exposed and opposite side of trephination was performed by using electric cranial drill. Then, the stimulating electrode was inserted into motor area of cerebral cortex. Meanwhile, the recording electrode was attached to sciatic nerve. To induce motor-evoked potentials (MEP), a single square wave (10 mA, 1 Hz) was applied. Recording data was imported into Origin64 to analyze amplitudes and latency.

### 2.22 Hot plate tests

Hot plate test was performed according to the previous study [27]. In brief, the hot plate was pre-heated, the temperature of hot plate was kept at 55 °C constantly. Mice were put on the surface of the hot plate. The time of first behavioral response was recorded.

### 2.23 Flow cytometry

Cells were collected and washed twice with PBS. After centrifugation, cells were incubated with F4/80 (Biolegend, 123137) and CD86 (Biolegend, 105007) antibodies at 4 °C in the dark for 1 h. Following this, cells were centrifuged again and washed twice with PBS. Before staining for CD206, cells were fixed and permeabilized with fixation/permeabilization kit (BD, 554714) at 4 °C in the dark for 20 min, then cells were incubated with the CD206 antibody (Biolegend, 141707) in the dark for 1 h. Using Flowjo (v10.9) to analyze the mean fluorescence intensity of CD86 and CD206 for each group separately.

### 2.24 Extraction of nuclear protein

Nuclear and Cytoplasmic Protein Extraction Kit (Beyotime, P0027) was utilized for the extraction of nuclear proteins, all steps were carried out according to the manual. To put it simply, cells were washed with PBS and scraped off with cell scrapers, the cytoplasmic proteins were initially separated using the cytoplasmic protein extraction reagent. The cytoplasmic fraction was then discarded, and the nuclear protein extraction reagent was added. The mixture was vortexed vigorously for 15 s every 2 min for a total of 30 min. After centrifugation, the supernatant collected consists of nuclear proteins.

### 2.25 Statistical analysis

The data for each group were normalized using the mean value of replicates from the Control group or the Sham group. All in vitro experiments were conducted with three biological replicates and more than two independent experiments, and all data were presented as the mean ± standard deviation (SD). The data were analyzed using one-way ANOVA in three groups and a two-tailed unpaired Student's t-test between two groups. The experimental data analysis was using GraphPad Prism 9 software, while the high-throughput sequencing data was analyzed using the R programming language (v.4.2.3). The statistical differences were considered at *p* < 0.05. ∗∗∗∗p < 0.0001, ∗∗∗p < 0.001, ∗∗p < 0.01, ∗p < 0.05. ns, no statistical significance.

## 3 Results

### 3.1 Establishment of the OGD/R model in HT22 cell line

We employed the OGD/R model to mimic the pathological process of ischemia-reperfusion in SCI. The OGD/R model in the mouse neuronal cell line HT22 cells was commonly used to study neuronal death following SCI. Following OGD/R treatment, a significant proportion of HT22 cells exhibited cellular pyknosis and lacked normal cellular structure (Fig. S1A). Additionally, the viability of the cells decreased (Fig. S1B), and Calcein-AM/PI double staining revealed an increased rate of cell death in the OGD/R group (Fig. S1C and D). Given the essential role of mitochondria in oxidation reactions and energy metabolism [28,29], mitochondrial dysfunction caused excessive ROS. This excess of ROS resulted in DNA damage and inhibited essential metabolic enzymes, eventually triggering cell death. JC-1 staining was employed to assess alterations in mitochondrial membrane potential (MMP). In cells with high MMP, JC-1 aggregates emitted red fluorescence, whereas a shift from red to green fluorescence indicated a reduction in MMP [30]. The results showed the ratio of aggregates/monomers was lower in OGD/R group, which indicated MMP decreased obviously after OGD/R treatment (Fig. S1E and F). We also assessed the ROS level of HT22 cells in OGD/R model, the results demonstrated that OGD/R indeed led to a significant production of ROS (Fig. S1G and H). The results of JC-1 staining and ROS detection indicate that mitochondrial function was impaired after OGD/R. The above results indicated that OGD/R model effectively simulated ischemia-reperfusion process of spinal cord injury.

### 3.2 ISRIB suppresses neuronal cell apoptosis induced by OGD/R

**Fig. 1:**
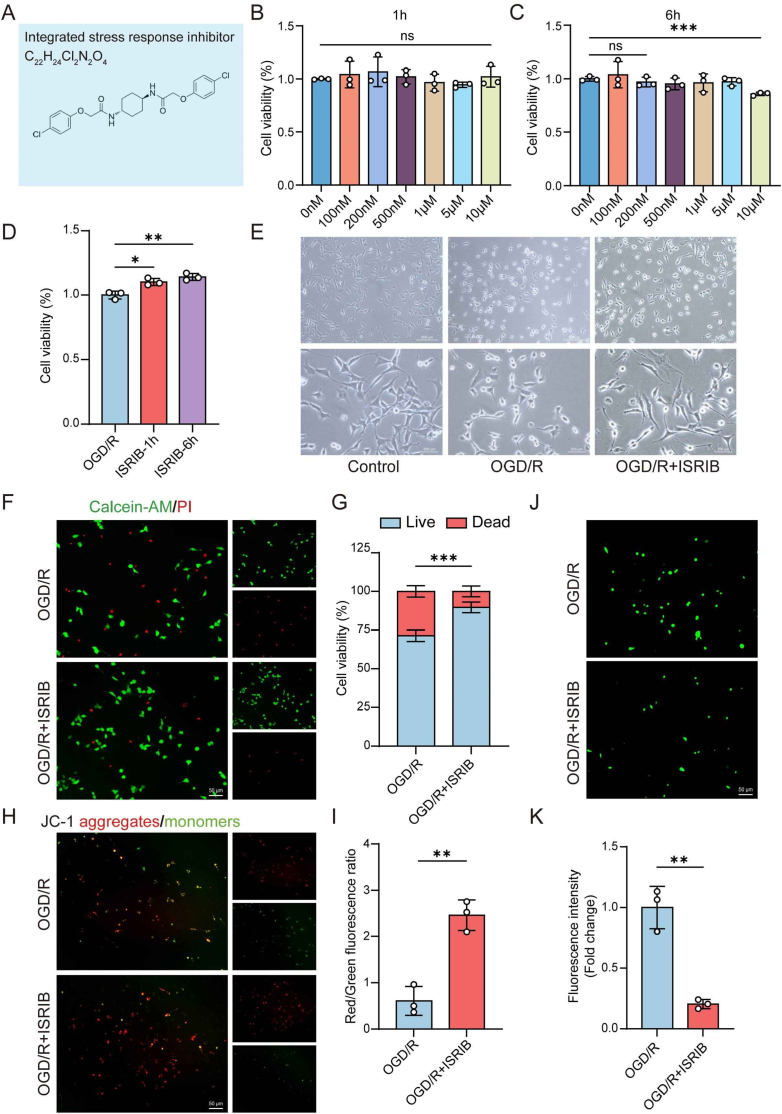
ISRIB suppresses neuronal cell apoptosis induced by OGD/R. (A) The chemical structure of ISRIB. (B–C) Cell viability of HT22 cells after treatment with different concentrations of ISRIB for 1 h (B) and 6 h (C). (D) Cell viability of HT22 cells pretreated with ISRIB at 200 nM concentrations for 1 h or 6 h following OGD/R. (E) Morphological changes were observed under light microscopy in HT22 of OGD/R and ISRIB groups. Scale bar of the first line: 500 μm, scale bar of the second line: 200 μm. (F–G) Live cells were determined through Calcein-AM/PI staining. Scale bar: 50 μm. (H–I) JC-1 staining was used to measure MMP of cells. Scale bar: 50 μm. (J–K) Intracellular ROS levels were measured with DCFH-DA staining of different groups. Scale bar: 50 μm. The data are presented as the means ± SD, n = 3. ∗p < 0.05, ∗∗p < 0.01, ∗∗∗p < 0.001.

ISRIB is a small molecule with a molecular weight of 451.3 g/mol and the chemical formula C_22_H_24_Cl_2_N_2_O_4_ (Fig. 1A). We used ISRIB to pretreat HT22 cells with the intention of protective effects. To determine the optimal conditions, we tested various ISRIB concentrations and treatment durations to evaluate its impact on cell viability. The results indicated 1 h treatment with ISRIB at concentrations ranging from 0 nM to 10 μM had no significant effect on cell viability (Fig. 1B). However, when HT22 cells were treated with 10 μM ISRIB for 6 h, cell viability decreased significantly. Based on previous studies where 200 nM ISRIB was commonly used in cellular experiments without affecting cell viability [31,32] (Fig. 1C), we selected 200 nM ISRIB for further studies. Both the 1 h and 6 h pretreatments with 200 nM ISRIB enhanced cell viability after OGD/R treatment, while the 6 h pretreatment demonstrating a more substantial protective effect (Fig. 1D). Thus, we chose to pretreat HT22 cells with 200 nM ISRIB for 6 h in subsequent experiments.

We found ISRIB partially alleviated the morphological changes induced by OGD/R in HT22 cells under light microscopy (Fig. 1E). The result of Calcein-AM/PI double staining showed a higher percentage of Calcein-AM positive cells in the ISRIB group, confirming that ISRIB reduced cell death in HT22 cells induced by OGD/R (Fig. 1F and G). Additionally, JC-1 staining showed the ratio of aggregates/monomers was higher in ISRIB group, which meant HT22 cells treated with ISRIB had higher MMP (Fig. 1H and I). ISRIB also effectively regulated the production of ROS, as evidenced by the lower intracellular ROS levels observed in the OGD/R + ISRIB group (Fig. 1J and K). The results strongly suggested that ISRIB exerted a protective effect on mitochondrial function. To extend these findings, we also examined the protective effect of ISRIB on primary cortical neurons in OGD/R model. Pretreating neurons with 200 nM ISRIB for 6 h before OGD/R treatment resulted in reduced neuronal death, as shown by Calcein-AM/PI staining (Fig. S2A and B). CCK-8 and JC-1 staining confirmed that ISRIB treatment improved cell viability (Fig. S2C) and increased MMP (Fig. S2D and E) compared to OGD/R group. Collectively, these results suggested that ISRIB could effectively mitigate neuronal damage induced by OGD/R.

### 3.3 ISRIB mitigates neuronal apoptosis through regulation of the P53 signaling pathway

**Fig. 2:**
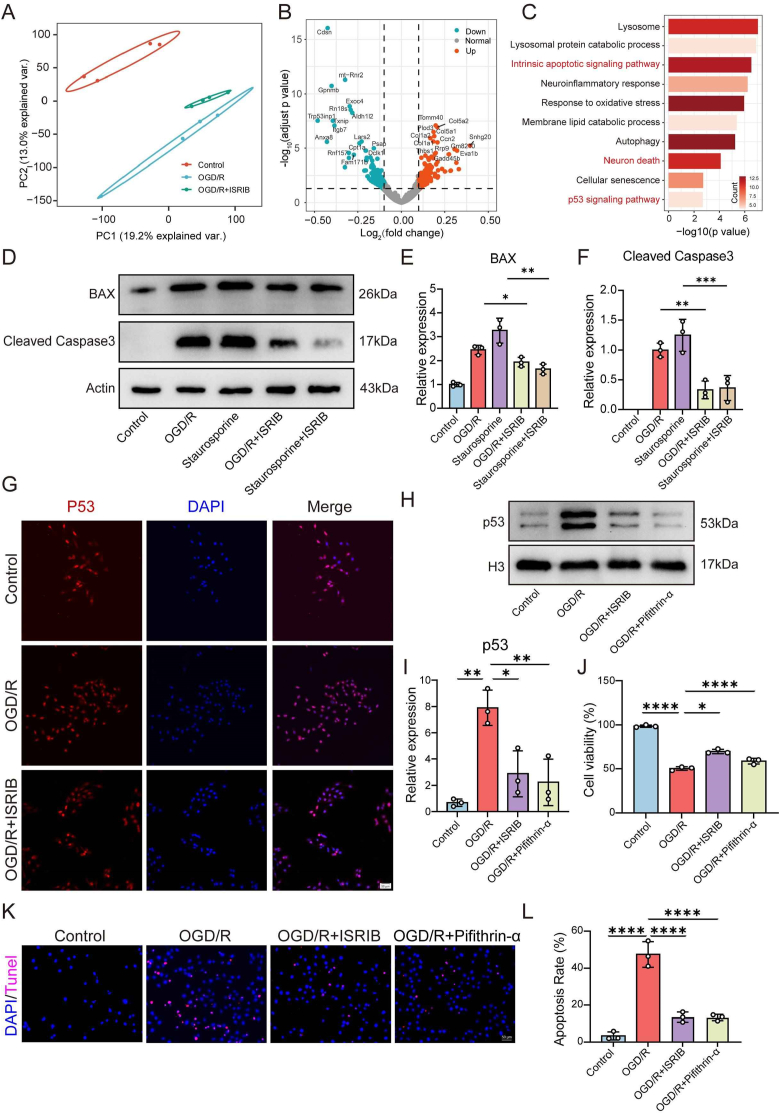
ISRIB mitigates neuronal apoptosis through regulation of the P53 signaling pathway. (A) PCA analysis of Control, OGD/R and OGD/R + ISRIB groups. (B)Volcano plot showed the differentially expressed genes between the OGD/R and OGD/R + ISRIB groups. (C)The functional enrichment of down-regulated genes in OGD/R and OGD/R + ISRIB groups. (D–F) The expression of BAX and cleaved Caspase3 in different groups. (G) Representative immunofluorescence images of P53 (red) and DAPI (blue) in HT22 cells among three groups. Nuclei were stained with DAPI to indicate the P53 located outside or within the nuclei. Scale bar:50 μm. (H–I) The expression of P53 and histone H3 in nuclei of HT22 cells. (J) Cell viability was detected in each group. (K–L) The percentage of apoptotic HT22 cells was detected by TUNEL staining. Scale bar: 50 μm. Data analysis was performed by One-way ANOVA. The data are presented as the means ± SD, n = 3. ∗p < 0.05, ∗∗p < 0.01, ∗∗∗p < 0.001, ∗∗∗∗p < 0.0001. ns, no significance.

To uncover the mechanism by which ISRIB reduces neuronal apoptosis, we used RNA-seq to detect changes in transcription levels of HT22 cells, including Control, OGD/R and OGD/R + ISRIB groups. Principal component analysis (PCA) showed that the biological replicates of the three groups were in different areas, indicating reliable inter-group differences in our data (Fig. 2A). Next, we compared the differentially expressed genes between the OGD/R + ISRIB and OGD/R groups, and identified that the down-regulated genes in the OGD/R + ISRIB group were closely related to apoptosis (such as Trp53inp1, Cdsn, etc.) (Fig. 2B). Functional enrichment analysis confirmed that these down-regulated genes were involved in cell death-related signaling pathways, such as “Intrinsic apoptotic signaling pathway” and “Neuron death”. Interestingly, the upstream regulatory pathway of apoptosis, p53 signaling pathway, was also enriched in results (Fig. 2C). To validate these observations, HT22 cells were pretreated with 200 nM ISRIB for 6 h, followed by either OGD/R treatment or apoptosis induction with 1 μM Staurosporine for 12 h. Western blot showed a significant increase in the apoptotic proteins BAX and cleaved Caspase3 after OGD/R treatment, but their expression was markedly reduced in the OGD/R + ISRIB group, indicating that ISRIB mitigated OGD/R-induced neuronal apoptosis. Similarly, in the Staurosporine induced apoptosis model, ISRIB reduced BAX and cleaved Caspase3 expression, further confirming its anti-apoptotic effects (Fig. 2D–F).

To delve deeper into the mechanism, we examined the subcellular localization of the P53 protein using immunofluorescence (Fig. 2G). The fluorescence results demonstrated that in the OGD/R group, P53 was predominantly localized in the nucleus. However, in the OGD/R + ISRIB group, its distribution was observed to be similar to that of the Control group, most accumulated in the nucleus, a portion situated outside the nucleus. This finding suggested that ISRIB inhibited the nuclear translocation of P53 in neuronal cells under OGD/R conditions. 10 μM Pifithrin-α was used to treat HT22 cells for 2 h before OGD/R [33], Western blot of nuclear proteins showed that both Pifithrin-α and ISRIB reduced nuclear P53 expression compared to the OGD/R group (Fig. 2H and I). Additionally, CCK-8 assays and TUNEL staining indicated that Pifithrin-α increased cell viability and reduced the percentage of TUNEL^+^ cells (Fig. 2J–L), demonstrating its effectiveness in decreasing neuronal apoptosis. These results further supported that ISRIB's protective effect is mediated through the inhibition of the P53 signaling pathway.

In vivo, neuronal apoptosis following SCI was assessed by examining cleaved Caspase-3 expression and TUNEL staining. The SCI + Vehicle (mixture of DMSO and PEG400) group exhibited a marked increase in cleaved Caspase-3 expression, whereas ISRIB treatment notably reduced its levels (Fig. S3A and C). The proportion of NeuN+/TUNEL + cells was higher in the SCI + Vehicle group compared to the Sham group but was decreased in mice treated with ISRIB (Fig. S3E). We also examined P53 expression in the spinal cord. Results showed that P53 expression was upregulated after SCI, and ISRIB reduced P53 expression compared to the SCI + Vehicle group (Fig. S3B and D). Overall, these findings demonstrated that ISRIB effectively reduced the nuclear translocation of P53, thereby alleviating neuronal apoptosis both in vitro and in vivo following SCI.

### 3.4 ISRIB reduces the differentiation of M1-type microglial cells

**Fig. 3:**
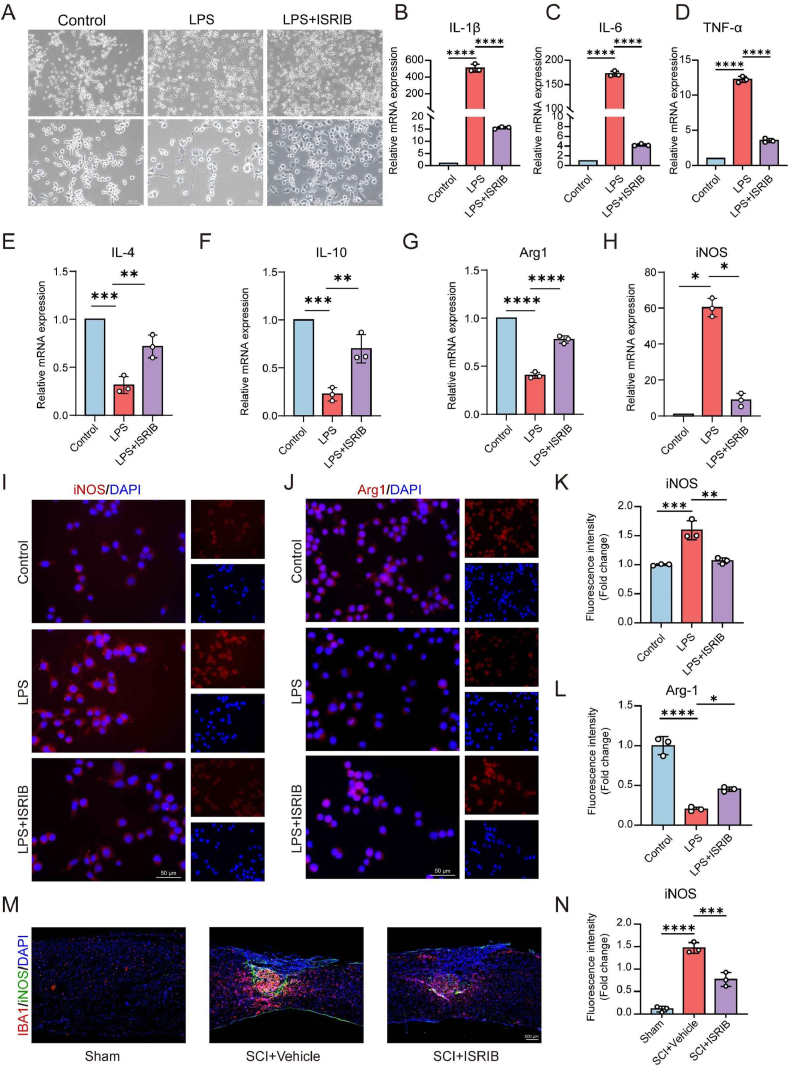
ISRIB reduces the differentiation of M1-type microglial cells induced by LPS. (A) Morphological changes were observed under light microscopy in BV2 cells of Control, LPS and LPS + ISRIB groups. Scale bar of the first line: 500 μm, scale bar of the second line: 200 μm. (B–H) The mRNA expression of IL-1β (B), IL-6 (C) and, TNF-α(D), IL-4(E), IL-10(F), Arg1(G) and iNOS(H) in Control, LPS and LPS + ISRIB groups. β-Actin was used as control. (I) The expression of iNOS(red) and DAPI (blue) in BV2 cells among three groups detected by immunofluorescence. Scale bar: 50 μm. (J) The expression of Arg1 (red) and DAPI (blue) in BV2 cells among three groups. Scale bar: 50 μm. (K) The fluorescence intensity of iNOS in Control, LPS and LPS + ISRIB groups. (L) The fluorescence intensity of Arg1 in Control, LPS and LPS + ISRIB groups. (M) The expression of IBA1 (red), iNOS(green) and DAPI (blue) in spinal cord tissues among three groups. (N) The fluorescence intensity of iNOS in Sham, SCI + Vehicle and SCI + ISRIB groups. Scale bar: 500 μm. Data analysis was performed by One-way ANOVA. The data are presented as the means ± SD, n = 3. ∗p < 0.05, ∗∗p < 0.01, ∗∗∗p < 0.001, ∗∗∗∗p < 0.0001.

The mouse microglial cell line BV2 was utilized in vitro studies of neuroinflammation after SCI. We established a neuroinflammation model by treating BV2 cells with 1 μg/ml LPS for 6 h [34]. After this, the LPS group was switched to medium alone, while the ISRIB group received medium with 200 nM ISRIB for the next 24 h. LPS treatment led to the formation of long protrusions and an ameboid-like shape in BV2 cells, which ISRIB partially mitigated (Fig. 3A). We analyzed the mRNA levels of pro-inflammatory factors (IL-1β, IL-6, and TNF-α) and anti-inflammatory factors (IL-4 and IL-10) in the Control, LPS, and LPS + ISRIB groups (Fig. 3B–F). LPS increased the mRNA levels of IL-1β, IL-6, and TNF-α, but ISRIB effectively reduced these pro-inflammatory cytokines. Conversely, while LPS decreased IL-4 and IL-10 levels, ISRIB significantly increased their expression. To explore impact of the ISRIB on BV2 cell polarization, we measured the M1 phenotype marker iNOS and the M2 marker Arg1 using qPCR and immunofluorescence (Fig. 3G–L) [35]. The LPS + ISRIB group showed lower iNOS expression and higher Arg1 levels compared to the LPS group. Flow cytometry confirmed these results, revealing that ISRIB reversed the LPS induced increase in CD86 and decrease in CD206 (Fig. S4A–D). Additionally, treating BV2 cells with 20 ng/ml recombinant murine IL-4 for 24 h, followed by 200 nM ISRIB for another 24 h, further enhanced M2 polarization, as indicated by elevated Arg1 levels (Fig. S4E).

Finally, we assessed microglial polarization after SCI by co-staining for IBA1 and iNOS. The results showed that ISRIB reduced M1 polarization of microglia (Fig. 3M and N). We also evaluated IL-1β expression in vivo and found lower levels in SCI + ISRIB group than SCI + Vehicle group. This suggested that ISRIB effectively suppressed post-injury inflammation in the spinal cord (Fig. S4K and O).

### 3.5 ISRIB inhibits immune-inflammatory responses by modulating the JAK2/STAT3 signaling pathway

**Fig. 4:**
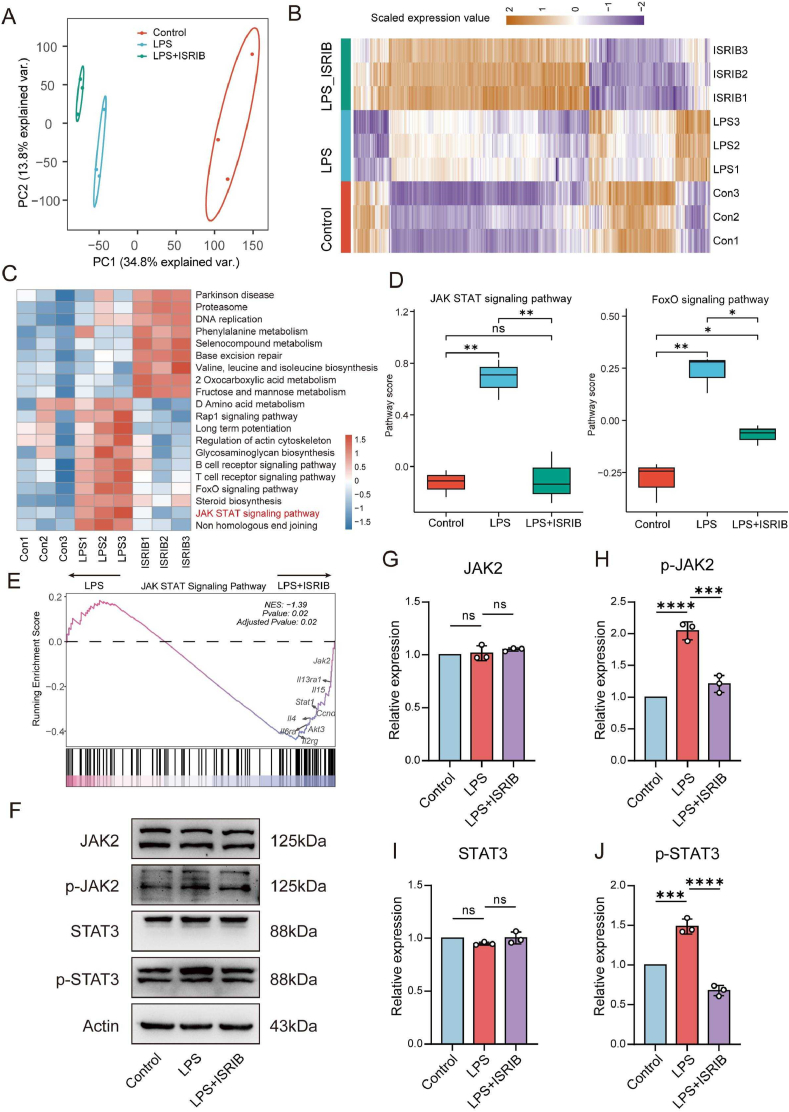
ISRIB inhibits immune-inflammatory responses by modulating the JAK2/STAT3 signaling pathway. (A) PCA analysis in Control, LPS and LPS + ISRIB groups. (B) Heatmap of differentially expressed genes in each group. (C) Heatmap showed the representative pathway terms of KEGG enriched in each cellular subgroup. (D) Boxplots showed JAK-STAT and FoxO signaling pathways among different groups. (E) GSEA of JAK-STAT signaling in the LPS + ISRIB and LPS group. (F–J) WB showed the expression of proteins in different groups (n = 3). Data analysis was performed by One-way ANOVA. The data are presented as the means ± SD. ∗p < 0.05, ∗∗p < 0.01, ∗∗∗p < 0.001, ∗∗∗∗p < 0.0001. ns, no significance.

Intrigued by the mechanism through which ISRIB reduced the generation of M1 microglial cells and inflammatory responses induced by LPS, we performed RNA sequencing on BV2 cells from Control, LPS, and LPS + ISRIB groups. The clear separation of these three groups in the principal component analysis (PCA) space indicated the high reliability of our data (Fig. 4A). Subsequently, heatmap illustrated the genes with specific expression patterns across the different groups (Fig. 4B). To gain further insights into the changes in signaling pathways among the groups, we conducted gene set variation analysis (GSVA) to score KEGG pathways (Fig. 4C). We identified pathways that were specifically activated in the LPS group and the LPS + ISRIB group. The results revealed that the majority of pathways specifically activated in the LPS + ISRIB group were related to metabolic functions, while the pathways restored in the LPS + ISRIB group compared to the LPS group were associated with immune inflammation. Notably, these pathways included the JAK-STAT signaling pathway and its related pathways, such as the FoxO signaling pathway, which were of particular interest to us (Fig. 4D). Furthermore, using gene set enrichment analysis (GSEA), we confirmed the suppression of JAK-STAT signaling activity in the LPS + ISRIB group compared to the LPS group (Fig. 4E). Interestingly, a significant proportion of genes ranked lower were associated with the JAK2/STAT3 signaling pathway.

We further assessed the expression on key proteins within this pathway and found that ISRIB treatment notably reduced p-JAK2 and p-STAT3 induced by LPS (Fig. 4F–J). To validate these observations, BV2 cells were treated with 0.5 μM JAK2/STAT3 inhibitor Cucurbitacin I for 1 h before LPS exposure [36]. Both ISRIB and Cucurbitacin I effectively decreased the mRNA levels of IL-1β, IL-6, and TNF-α (Fig. S4F–H), reduced iNOS expression (Fig. S4I and M), and increased Arg1 expression (Fig. S4J and N) compared to the LPS group. These findings suggest a shift toward M2 polarization and highlight JAK2/STAT3 as a key pathway of anti-inflammatory effects.

Additionally, we assessed the expression of p-STAT3 in spinal cord. The increased expression of p-STAT3 in the SCI + Vehicle group compared to the Sham group was notably reduced by ISRIB treatment (Fig. S4L and P). Collectively, these results illustrated that ISRIB effectively modulated the JAK2/STAT3 signaling pathway to exert its anti-inflammatory effects both in vitro and in vivo.

### 3.6 ISRIB promotes function recovery after spinal cord injury

**Fig. 5:**
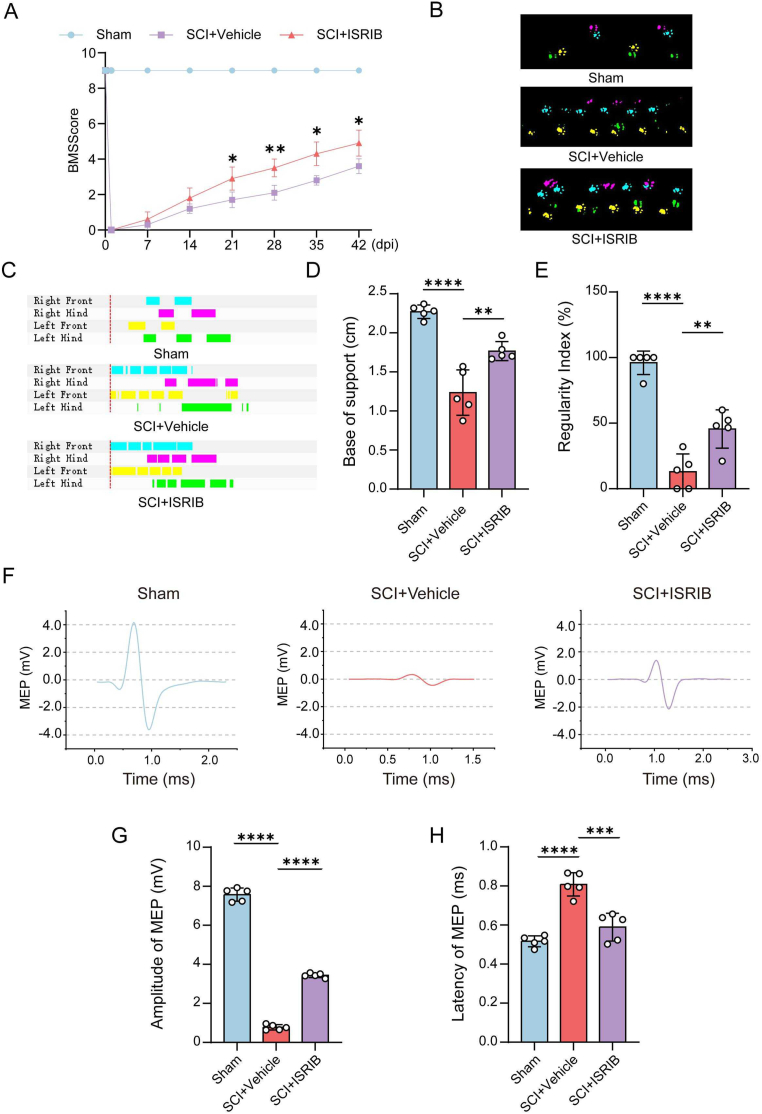
ISRIB promoted function recovery after spinal cord injury. (A) The BMS scores of mice at different time after SCI. (B–C) The footprints of Sham, SCI and SCI + ISRIB groups at 42 dpi. (D)Comparison base of support of hind limbs among three groups. (E) The regularity index of each group. (F–H) The quantification of Amplitude and Latency in each group at 6 weeks post SCI. Data analysis was performed by One-way ANOVA. The data are presented as the means ± SD, n = 5. ∗∗*p* < 0.01, ∗∗∗∗*p* < 0.0001.

In order to investigate the reparative effects of ISRIB on SCI, mice received intraperitoneal injections of ISRIB or vehicle for 28 days, followed by behavioral tests and spinal cord immunofluorescence at 42 dpi (Fig. S5A). HE staining showed that ISRIB had no obvious side effects (Fig. S5B). Notably, ISRIB treatment reduced the damage area compared to the SCI + Vehicle group, indicating its reparative effect on SCI (Fig. S5C). Behavioral assessments using BMS scores showed that ISRIB-treated mice had significantly higher scores from 21 dpi onward, reflecting improved hindlimb locomotor function (Fig. 5A). Footstep analysis at 42 dpi revealed an increased base of support and more coordinated footprints in the ISRIB group (Fig. 5B–E). Electrophysiological assessments supported these findings, with ISRIB-treated mice showing higher amplitude and reduced latency of motor evoked potential (MEP) compared to the SCI + Vehicle group (Fig. 5F–H). Additionally, ISRIB treatment improved bladder function, as indicated by a thicker bladder wall than SCI + Vehicle group (Fig. S5D). Sensory recovery was assessed using a hot plate test, which demonstrated increased sensitivity to thermal stimulation in the SCI + ISRIB group, suggesting enhanced sensory function (Fig. S5E). Overall, these results provide compelling evidence that ISRIB significantly promotes functional recovery in a mouse model of SCI.

## 4 Discussion

SCI poses a significant challenge to patient quality of life, marked by a complex cascade of events that extend beyond the initial trauma. The primary injury involves spinal cord compression and transection, resulting in neuronal death within the injured region and disruption of the blood-spinal cord barrier. These changes collectively contribute to the development of secondary injury. During the acute phase of SCI, blood vessel destruction leads to inadequate oxygen and nutrient supply. In the subacute phase, ischemia and excitotoxicity further disrupt cellular ionic homeostasis. The release of free radicals and glutamate from injured neurons accelerates neuronal cell death [37]. Despite the documentation of various types of cell death in SCI, apoptosis is generally considered to have a more significant impact on SCI repair. The significant impairment of neurological function following spinal cord injury is largely attributed to extensive neuronal apoptosis. Neuronal apoptosis occurs as early as 4 h following SCI and reaches its peak at 8 h after SCI [38,39]. Ischemia/reperfusion injury, a common feature in many neurological conditions, leads to extensive cell membrane and organelle damage, culminating in neuronal apoptosis [40,41].

Given the critical role of neurons in transmitting sensory and motor signals, preventing neuronal death is essential for functional recovery. Pharmacological interventions aimed at reducing neuronal death have emerged as a promising approach [42,43]. ISRIB is a small molecule compound capable of crossing the blood–brain barrier following intraperitoneal injection, allowing it to effectively reach the central nervous system. Its plasma half-life is approximately 8 h [24]. Previous research had concentrated on the function of ISRIB in regulating the integrated stress response [[44], [45], [46]]. However, it was also conceivable that ISRIB influenced neuronal death through alternative mechanisms. In vitro and in vivo experiments demonstrated that ISRIB mitigated neuronal apoptosis via the P53 signaling pathway. The reduction of P53 expression within the nuclei of HT22 cells was a critical factor in the observed decrease in apoptosis rates, a finding that was also confirmed in SCI mice. Therefore, the P53 signaling pathway emerged as a crucial target for intervention in neuronal apoptosis, with the potential to facilitate repair of SCI.

Inflammation constitutes a crucial component of secondary injury, playing a pivotal role in the progression of SCI and regulation of neuronal damage. Microglial cells promptly become activated in response to SCI and act as critical mediators of inflammation. During the early stages, microglia assume protective roles [47]. But microglia switch to pro-inflammatory cells rapidly. Microglia are active by Damage-Associated Molecular Patterns (DAMPs) [48]. Resident microglia and astrocytes released cytokines and chemokines within hours, IL-1β, IL-6 and TNF-α are the most important cytokines and studied deeply [15]. Cytokines and chemokines will attract microglia, peripherally derived macrophages infiltration. Activated M1 microglia in secondary injury contribute to neuronal cell death and release cytotoxic substances, thereby initiating a cascade of toxic reactions. In contrast, the M2 phenotype plays a role in dampening inflammation and promoting repair processes [49]. Many studies reported suppressing inflammation is helpful for function recovery in SCI. Alpinetin reduced cytokines release of microglia by inhibited JAK2/STAT3 pathway [50], Cinepazide maleate inhibited inflammation to improve recovery in SCI [51]. ISRIB has been shown to attenuate microglia infiltration by suppressing the integrated stress response in rat surgical brain injury. However, it remains unclear whether ISRIB can suppress inflammation in SCI. The findings of this study indicated that ISRIB had the effect of reducing the synthesis of pro-inflammatory factors and promoting the expression of anti-inflammatory factors. Furthermore, ISRIB effectively facilitated the transition of microglia from an M1 to an M2 polarization state. These findings suggested that ISRIB may have clinical applications in regulating neuroinflammation following SCI. The results suggested that the JAK2/STAT3 signaling pathway represented another mechanism through which ISRIB regulated neuroinflammation following SCI. Moreover, our findings substantiated the notion that the inhibition of JAK2 and STAT3 phosphorylation could effectively mitigate neuroinflammatory processes [52,53].

The treatment of SCI is a great challenge. Even no approach has been found to make spinal cord injured patient complete recovery, but previous studies have found some strategies to improve function recovery and enhance life qualities of patients. Pharmacological treatment is feasible in SCI. For example, corticosteroids are well-recognized for anti-inflammatory effect in SCI [54], gangliosides have neuroprotective effect [55,56]. However, the efficacy of certain drugs has been called into question, underscoring the urgent need for safer and more effective therapeutic options. Unlike other drugs that target SCI through a single pathway, ISRIB demonstrates dual mechanisms of action by both reducing neuronal apoptosis and modulating neuroinflammation. This dual functionality enhances ISRIB's potential as a comprehensive treatment for SCI, suggesting broad application prospects.

There are two limitations of this study. First, even though we did a lot of experiment in vitro and in mice SCI model, whether ISRIB could be used in clinic need to do some large animal experiment. Secondly, although we have identified potential signaling pathways through which ISRIB exerts its inhibitory effects on apoptosis and inflammation, the direct regulatory role of ISRIB on key proteins within these pathways remains a focus of our future research.

## 5 Conclusion

**Fig. 6:**
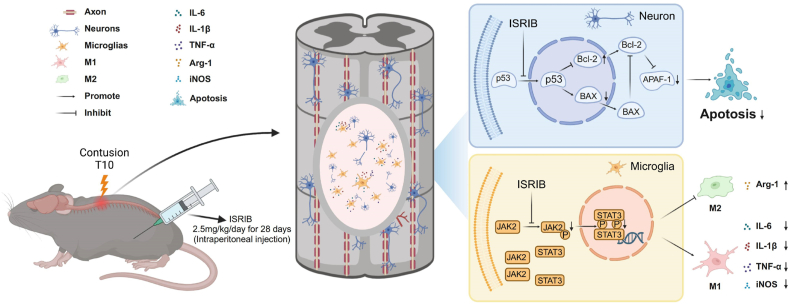
Schematic illustration for the mechanism of ISRIB in SCI. ISRIB decreased expression of P53 in nucleus to attenuate the neurons apoptosis and suppressed the phosphorylation of JAK2 and STAT3 to modulate neuroinflammation in SCI mice.

In conclusion, our findings indicated that ISRIB played a role in promoting the functional recovery of SCI through the regulation of neuronal apoptosis via the P53 signaling pathway and the modulation of neuroinflammation through the JAK2/STAT3 signaling pathway (Fig. 6). These observations suggested that ISRIB might serve as a potential therapeutic strategy for the repair of SCI in the future.

## Author contributions

QL, CZ and EQ performed the experiments and analyzed the data, they contributed equally to this study. SF, HZ (Hengxing Zhou) and HZ (Hua Zhao) designed and supervised the study. CZ and MW performed bioinformatic analysis. HS, TZ and YJ performed investigation. HL, RJ and CL checked the data. QL and CZ wrote the manuscript. All authors agreed to the published version of the manuscript.

## Funding

This work was funded by Taishan Scholars Program of Shandong Province-Young Taishan Scholars (tsqn201909197), Cutting Edge Development Fund of Advanced Medical Research Institute (Shandong University), 10.13039/501100007129Shandong Provincial Natural Science Foundation (ZR2021MH134) and 10.13039/501100002858China Postdoctoral Science Foundation (2019M652394).

## Declaration of competing interest

The authors have no conflicts of interest relevant to this article.

## References

[1] McDonald J.W., Sadowsky C. (2002). Spinal-cord injury. Lancet.

[2] Collaborators G.S.C.I. (2023). Global, regional, and national burden of spinal cord injury, 1990-2019: a systematic analysis for the Global Burden of Disease Study 2019. Lancet Neurol.

[3] Zhou H., Lou Y., Chen L., Kang Y., Liu L., Cai Z. (2024). Epidemiological and clinical features, treatment status, and economic burden of traumatic spinal cord injury in China: a hospital-based retrospective study. Neural Regen Res.

[4] Skeers P., Battistuzzo C.R., Clark J.M., Bernard S., Freeman B.J.C., Batchelor P.E. (2018). Acute thoracolumbar spinal cord injury. J Bone Joint Surg.

[5] Shin J.E., Jung K., Kim M., Hwang K., Lee H., Kim I.-S. (2018). Brain and spinal cord injury repair by implantation of human neural progenitor cells seeded onto polymer scaffolds. Exp Mol Med.

[6] Kitchen P., Salman M.M., Halsey A.M., Clarke-Bland C., MacDonald J.A., Ishida H. (2020). Targeting aquaporin-4 subcellular localization to treat central nervous system edema. Cell.

[7] Hutson T.H., Di Giovanni S. (2019). The translational landscape in spinal cord injury: focus on neuroplasticity and regeneration. Nat Rev Neurol.

[8] He Z., Du J., Zhang Y., Xu Y., Huang Q., Zhou Q. (2023). Kruppel-like factor 2 contributes to blood-spinal cord barrier integrity and functional recovery from spinal cord injury by augmenting autophagic flux. Theranostics.

[9] Smith PD, Puskas F, Meng X, Lee JH, Cleveland JC, Weyant MJ (2012). The evolution of chemokine release supports a bimodal mechanism of spinal cord ischemia and reperfusion injury. Circulation.

[10] Rong Y., Fan J., Ji C., Wang Z., Ge X., Wang J. (2021). USP11 regulates autophagy-dependent ferroptosis after spinal cord ischemia-reperfusion injury by deubiquitinating Beclin 1. Cell Death Differ.

[11] Yune T.Y., Lee J.Y., Jiang M.H., Kim D.W., Choi S.Y., Oh T.H. (2008). Systemic administration of PEP-1–SOD1 fusion protein improves functional recovery by inhibition of neuronal cell death after spinal cord injury. Free Radic Biol Med.

[12] Shi Z, Yuan S, Shi L, Li J, Ning G, Kong X (2021). Programmed cell death in spinal cord injury pathogenesis and therapy. Cell Prolif.

[13] Abbaszadeh F, Fakhri S, Khan H (2020). Targeting apoptosis and autophagy following spinal cord injury: therapeutic approaches to polyphenols and candidate phytochemicals. Pharmacol Res.

[14] Yao X., Sun C., Fan B., Zhao C., Zhang Y., Duan H. (2021). Neurotropin exerts neuroprotective effects after spinal cord injury by inhibiting apoptosis and modulating cytokines. J Orthop Translat.

[15] Hellenbrand DJ, Quinn CM, Piper ZJ, Morehouse CN, Fixel JA, Hanna AS (2021). Inflammation after spinal cord injury: a review of the critical timeline of signaling cues and cellular infiltration. J Neuroinflammation.

[16] Pineau I., Lacroix S. (2006). Proinflammatory cytokine synthesis in the injured mouse spinal cord: multiphasic expression pattern and identification of the cell types involved. J Comp Neurol.

[17] Wu H, Zheng J, Xu S, Fang Y, Wu Y, Zeng J (2021). Mer regulates microglial/macrophage M1/M2 polarization and alleviates neuroinflammation following traumatic brain injury. J Neuroinflammation.

[18] Sidrauski C, Acosta-Alvear D, Khoutorsky A, Vedantham P, Hearn BR, Li H (2013). Pharmacological brake-release of mRNA translation enhances cognitive memory. Elife.

[19] Krukowski K, Nolan A, Frias ES, Boone M, Ureta G, Grue K (2020). Small molecule cognitive enhancer reverses age-related memory decline in mice. Elife.

[20] Halliday M, Radford H, Sekine Y, Moreno J, Verity N, le Quesne J (2015). Partial restoration of protein synthesis rates by the small molecule ISRIB prevents neurodegeneration without pancreatic toxicity. Cell Death Dis.

[21] Chou A, Krukowski K, Jopson T, Zhu PJ, Costa-Mattioli M, Walter P (2017). Inhibition of the integrated stress response reverses cognitive deficits after traumatic brain injury. Proc Natl Acad Sci USA.

[22] Zhang L., Zhi K., Su Y., Peng W., Meng X. (2023). Effect of eIF2α in neuronal injury induced by high glucose and the protective mechanism of resveratrol. Mol Neurobiol.

[23] Zhang X., Han Y., Fan C., Jiang Y., Jiang W., Zheng C. (2024). Epigallocatechin gallate induces apoptosis in multiple myeloma cells through endoplasmic reticulum stress induction and cytoskeletal disruption. Int Immunopharm.

[24] Sidrauski C., Acosta-Alvear D., Khoutorsky A., Vedantham P., Hearn B.R., Li H. (2013). Pharmacological brake-release of mRNA translation enhances cognitive memory. Elife.

[25] Zhang C, Yi X, Hou M, Li Q, Li X, Lu L (2023). The landscape of m(1)A modification and its posttranscriptional regulatory functions in primary neurons. Elife.

[26] Ren J., Zhu B., Gu G., Zhang W., Li J., Wang H. (2023). Schwann cell-derived exosomes containing MFG-E8 modify macrophage/microglial polarization for attenuating inflammation via the SOCS3/STAT3 pathway after spinal cord injury. Cell Death Dis.

[27] Lopes B.C., Medeiros L.F., Silva de Souza V., Cioato S.G., Medeiros H.R., Regner G.G. (2020). Transcranial direct current stimulation combined with exercise modulates the inflammatory profile and hyperalgesic response in rats subjected to a neuropathic pain model: long-term effects. Brain Stimul.

[28] Hoye A.T., Davoren J.E., Wipf P., Fink M.P., Kagan V.E. (2008). Targeting mitochondria. Acc Chem Res.

[29] Cheng Z., Ristow M. (2013). Mitochondria and metabolic homeostasis. Antioxidants Redox Signal.

[30] Shen K, Wang X, Wang Y, Jia Y, Zhang Y, Wang K (2023). miR-125b-5p in adipose derived stem cells exosome alleviates pulmonary microvascular endothelial cells ferroptosis via Keap1/Nrf2/GPX4 in sepsis lung injury. Redox Biol.

[31] Sidrauski C, McGeachy AM, Ingolia NT, Walter P (2015). The small molecule ISRIB reverses the effects of eIF2α phosphorylation on translation and stress granule assembly. Elife.

[32] Young-Baird S.K., Lourenço M.B., Elder M.K., Klann E., Liebau S., Dever T.E. (2020). Suppression of MEHMO syndrome mutation in eIF2 by small molecule ISRIB. Mol Cell.

[33] Yu F.F., Yu S.Y., Sun L., Zuo J., Luo K.T., Wang M. (2024). T-2 toxin induces mitochondrial dysfunction in chondrocytes via the p53-cyclophilin D pathway. J Hazard Mater.

[34] Zusso M, Lunardi V, Franceschini D, Pagetta A, Lo R, Stifani S (2019). Ciprofloxacin and levofloxacin attenuate microglia inflammatory response via TLR4/NF-kB pathway. J Neuroinflammation.

[35] Zeng H, Liu N, Yang Y-y, Xing H-y, Liu X-x, Li F (2019). Lentivirus-mediated downregulation of α-synuclein reduces neuroinflammation and promotes functional recovery in rats with spinal cord injury. J Neuroinflammation.

[36] Wang X., Li X., Zuo X., Liang Z., Ding T., Li K. (2021). Photobiomodulation inhibits the activation of neurotoxic microglia and astrocytes by inhibiting Lcn2/JAK2-STAT3 crosstalk after spinal cord injury in male rats. J Neuroinflammation.

[37] Emery E., Aldana P., Bunge M.B., Puckett W., Srinivasan A., Keane R.W. (1998). Apoptosis after traumatic human spinal cord injury. J Neurosurg.

[38] Beattie M.S., Farooqui A.A., Bresnahan J.C. (2000). Review of current evidence for apoptosis after spinal cord injury. J Neurotrauma.

[39] Bertheloot D., Latz E., Franklin B.S. (2021). Necroptosis, pyroptosis and apoptosis: an intricate game of cell death. Cell Mol Immunol.

[40] Li R., Zhao K., Ruan Q., Meng C., Yin F. (2020). The transcription factor Foxd3 induces spinal cord ischemia-reperfusion injury by potentiating microRNA-214-dependent inhibition of Kcnk2. Exp Mol Med.

[41] Cai Y, Yang E, Yao X, Zhang X, Wang Q, Wang Y (2021). FUNDC1-dependent mitophagy induced by tPA protects neurons against cerebral ischemia-reperfusion injury. Redox Biol.

[42] Xiao S, Zhong N, Yang Q, Li A, Tong W, Zhang Y (2022). Aucubin promoted neuron functional recovery by suppressing inflammation and neuronal apoptosis in a spinal cord injury model. Int Immunopharmacol.

[43] Pei J-p, Fan L-h, Nan K, Li J, Dang X-q, Wang K-z (2017). HSYA alleviates secondary neuronal death through attenuating oxidative stress, inflammatory response, and neural apoptosis in SD rat spinal cord compression injury. J Neuroinflammation.

[44] Goswami P., Akhter J., Mangla A., Suramya S., Jindal G., Ahmad S. (2024). Downregulation of ATF-4 attenuates the endoplasmic reticulum stress-mediated neuroinflammation and cognitive impairment in experimentally induced Alzheimer's disease model. Mol Neurobiol.

[45] Zyryanova A.F., Kashiwagi K., Rato C., Harding H.P., Crespillo-Casado A., Perera L.A. (2021). ISRIB blunts the integrated stress response by allosterically antagonising the inhibitory effect of phosphorylated eIF2 on eIF2B. Mol Cell.

[46] Anand A.A., Walter P. (2020). Structural insights into ISRIB, a memory-enhancing inhibitor of the integrated stress response. FEBS J.

[47] Hines D.J., Hines R.M., Mulligan S.J., Macvicar B.A. (2009). Microglia processes block the spread of damage in the brain and require functional chloride channels. Glia.

[48] Gong T., Liu L., Jiang W., Zhou R. (2019). DAMP-sensing receptors in sterile inflammation and inflammatory diseases. Nat Rev Immunol.

[49] Orihuela R., McPherson C.A., Harry G.J. (2015). Microglial M1/M2 polarization and metabolic states. Br J Pharmacol.

[50] Xiao S., Zhang Y., Liu Z., Li A., Tong W., Xiong X. (2023). Alpinetin inhibits neuroinflammation and neuronal apoptosis via targeting the JAK2/STAT3 signaling pathway in spinal cord injury. CNS Neurosci Ther.

[51] Li D., Zhao S., Zhu B., Zhao W., Ding Y., Li X. (2023). Cinepazide maleate promotes recovery from spinal cord injury by inhibiting inflammation and prolonging neuronal survival. Drug Dev Res.

[52] Zhu H., Jian Z., Zhong Y., Ye Y., Zhang Y., Hu X. (2021). Janus kinase inhibition ameliorates ischemic stroke injury and neuroinflammation through reducing NLRP3 inflammasome activation via JAK2/STAT3 pathway inhibition. Front Immunol.

[53] Zhou Q., Lin L., Li H., Wang H., Jiang S., Huang P. (2021). Melatonin reduces neuroinflammation and improves axonal hypomyelination by modulating M1/M2 microglia polarization via JAK2-STAT3-telomerase pathway in postnatal rats exposed to lipopolysaccharide. Mol Neurobiol.

[54] Kwon B (2004). Pathophysiology and pharmacologic treatment of acute spinal cord injury. Spine J.

[55] Chinnock P, Roberts I (2005). Gangliosides for acute spinal cord injury. Cochrane Database Syst Rev.

[56] Constantini S., Young W. (1994). The effects of methylprednisolone and the ganglioside GM1 on acute spinal cord injury in rats. J Neurosurg.

