# Supplementary material for: ISRIB facilitates post-spinal cord injury recovery through attenuation of neuronal apoptosis and modulation of neuroinflammation
Source: J Orthop Translat. 2025 Mar 7;51:119–31. doi: 10.1016/j.jot.2025.01.003 (PMC11930150; doi:10.1016/j.jot.2025.01.003)
Supplement: Multimedia component 1 [file mmc1.docx]

**Appendix A. Supplementary data**


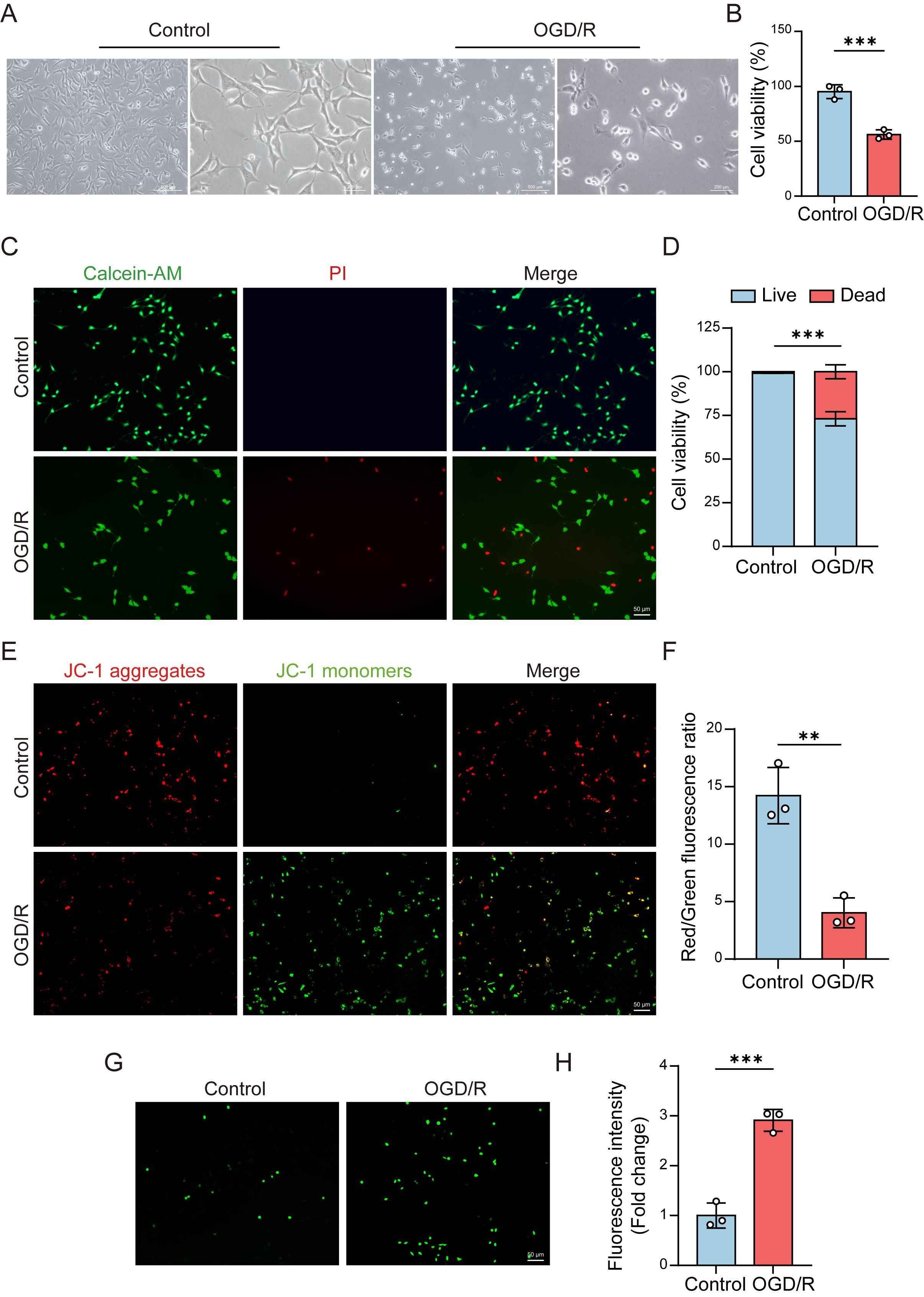


**Fig.S1. Establishment of the OGD/R model in HT22 cell line.** (A) Cellular morphology was observed under light microscopy. Scale bar of Control and OGD/R: 500μm(left), scale bar of Control and OGD/R: 200μm(right). (B) Cell viability in Control and OGD/R groups. (C-D) Calcein-AM/PI staining in Control and OGD/R groups. Scale bar: 50μm. (E-F) MMP of HT22 cells were measured with JC-1 staining. Scale bar: 50μm. (G-H) Intracellular ROS levels of Control and OGD/R groups were evaluated in HT22 cells. Scale bar: 50μm. Data analysis was performed by Student’s t-test. The data are presented as the means ± SD, n = 3. ***p*<0.05, ****p* < 0.001.


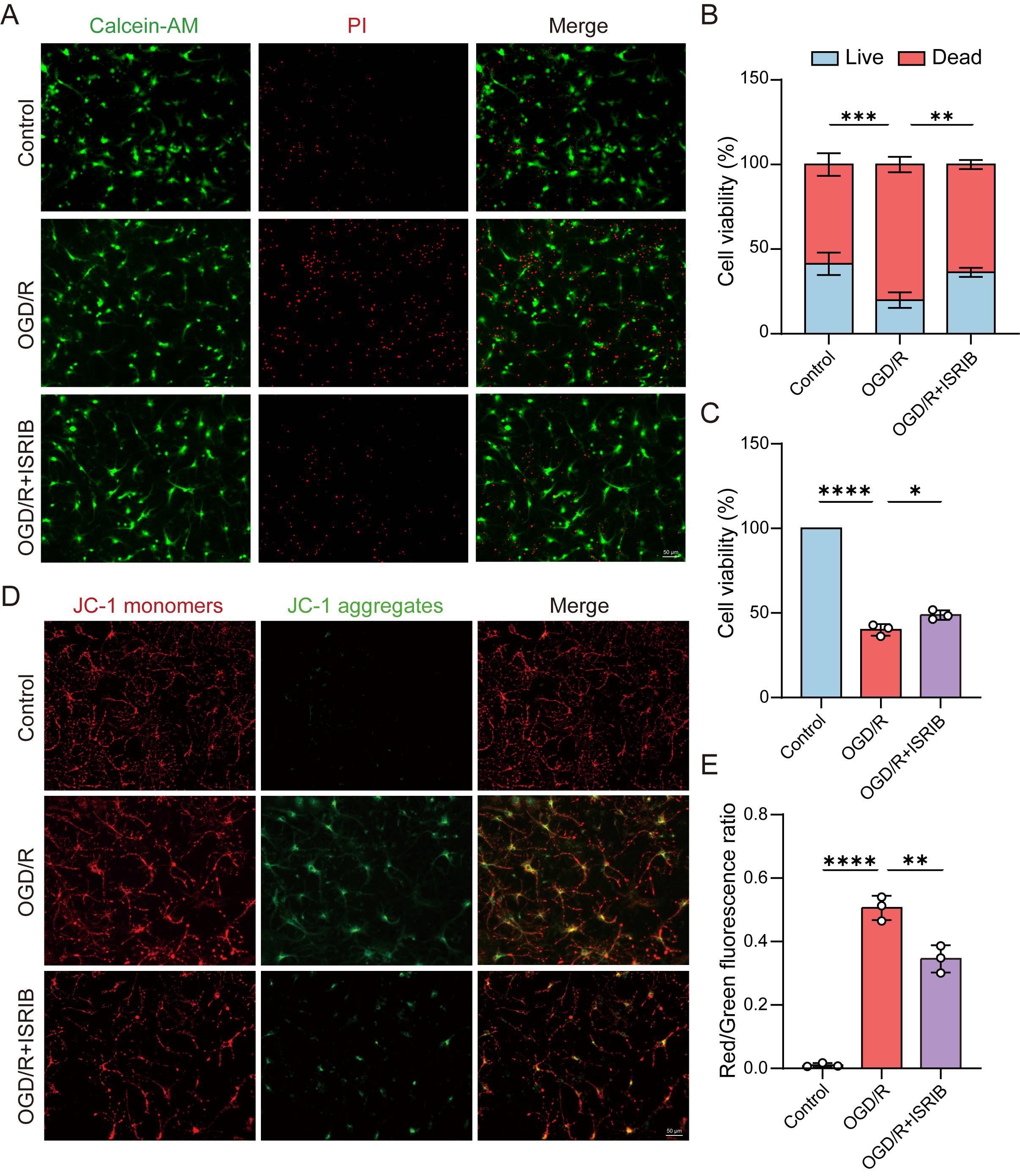


**Fig.S2. ISRIB suppresses neuronal cell apoptosis induced by OGD/R. (**A-B) Calcein-AM/PI staining was performed in primary cortical neurons. Scale bar: 50μm. (C) CCK-8 assays were used to assess cell in primary cortical neurons. (D-E) JC-1 staining was used to measure MMP in primary cortical neurons. Scale bar: 50μm. Data analysis was performed by One-way ANOVA. The data are presented as the means ± SD, n=3. *p < 0.05, **p < 0.01, ***p < 0.001, ****p < 0.0001.


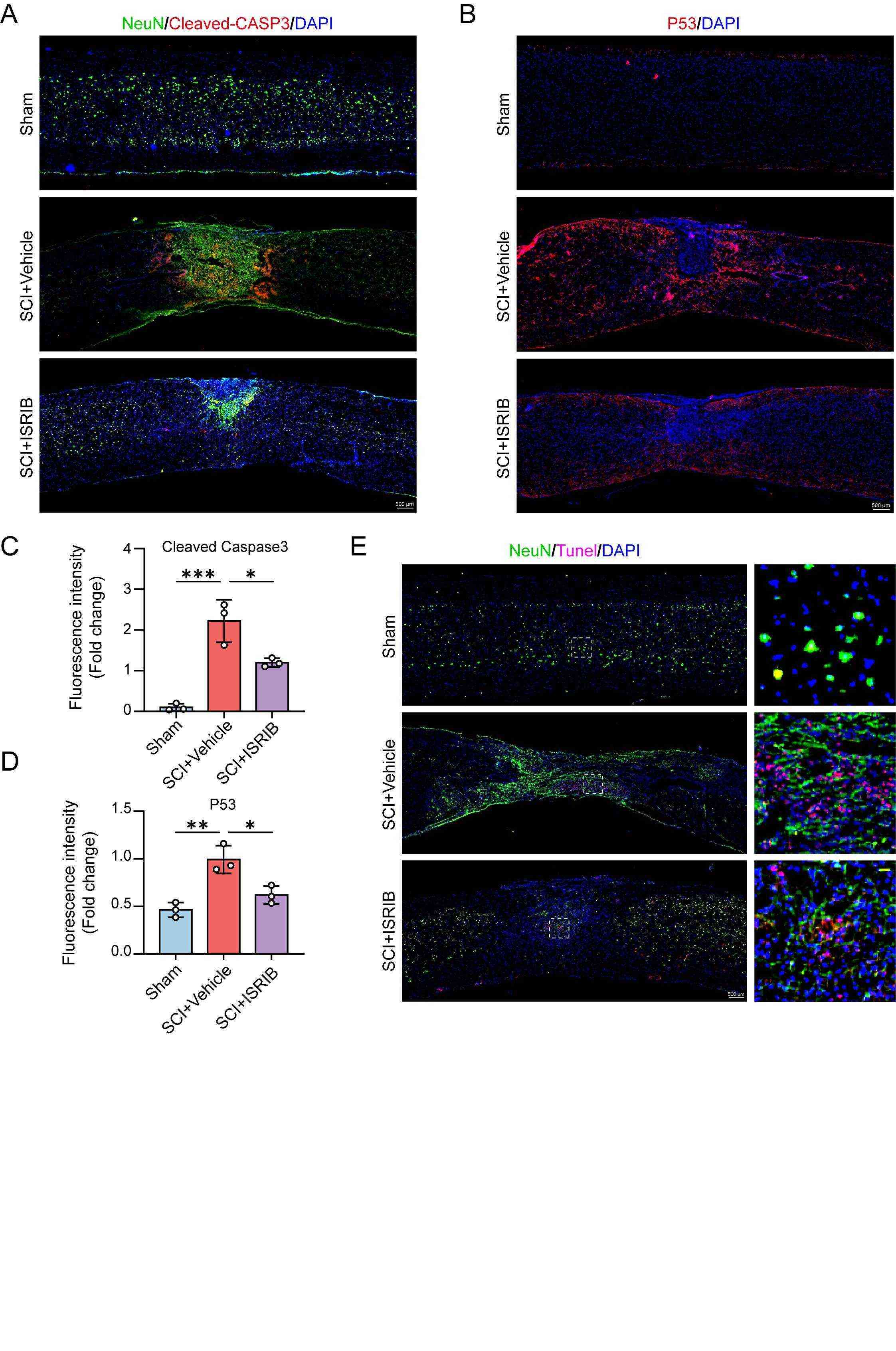


**Fig. S3. ISRIB mitigates neuronal apoptosis through regulation of the P53 signaling pathway.** (A) Immunofluorescence of NeuN (green), cleaved Caspase3(red) and DAPI (blue) in spinal cord tissues among three groups. Scale bar: 500μm. (B) Immunofluorescence of P53(red) and DAPI (blue) in spinal cord tissues among three groups. Scale bar: 500μm. (C) The fluorescence intensity of cleaved Caspase3 in Sham, SCI+Vehicle and SCI+ISRIB groups. (D) The fluorescence intensity of P53 in Sham, SCI+Vehicle and SCI+ISRIB groups. (E) Immunofluorescence of NeuN (green), TUNEL (red) and DAPI (blue) in spinal cord tissues among three groups. Scale bar: 500μm.The data are presented as the means ± SD. *p < 0.05, **p < 0.01, ***p < 0.001, n=3.


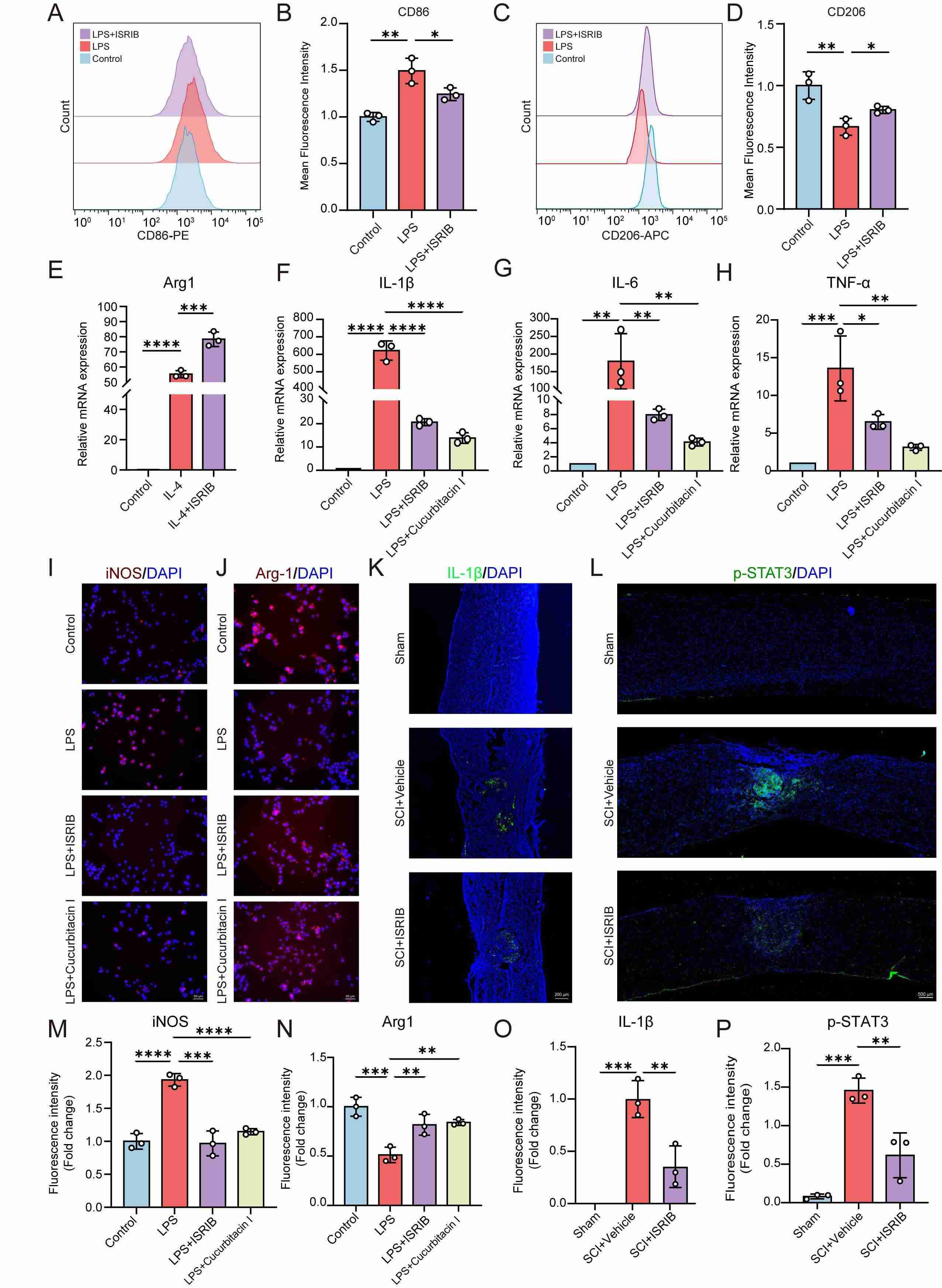


**Fig. S4.** **ISRIB reduces the differentiation of M1-type microglial cells.** (A-B) The expression of CD86 in each group detected by flow cytometry. (C-D) The expression of CD206 in each group detected by flow cytometry. (E) The mRNA expression of Arg1 in Control, IL-4, and IL-4+ISRIB groups. (F-H) The mRNA expression of IL-1β (F), IL-6 (G) and TNF-α (H) in different groups. (I) Representative immunofluorescence images of iNOS (red) and DAPI (blue) in different groups. Scale bar:50 μm. (J) Representative immunofluorescence images of Arg1 (red) and DAPI (blue) in different groups. Scale bar:50 μm. (K) Representative immunofluorescence images of IL-1β (green) and DAPI (blue) in the injured area of the spinal cord. Scale bar:200 um. (L) The detection of p-STAT3 expression in spinal cord tissue of each group by Immunofluorescence. Scale bar:500 μm. (M) The fluorescence intensity of iNOS in Control, LPS, LPS+ISRIB and LPS+Cucurbitacin I groups. (N) The fluorescence intensity of Arg1 in Control, LPS, LPS+ISRIB and LPS+Cucurbitacin I groups. (O) The fluorescence intensity of IL-1β in Sham, SCI+Vehicle and SCI+ISRIB groups. (P) The fluorescence intensity of p-STAT3 in Sham, SCI+Vehicle and SCI+ISRIB groups. Data analysis was performed by One-way ANOVA. The data are presented as the means ±SD, n=3. **p* < 0.05, ***p* < 0.01, ****p* < 0.001, *****p* < 0.0001.


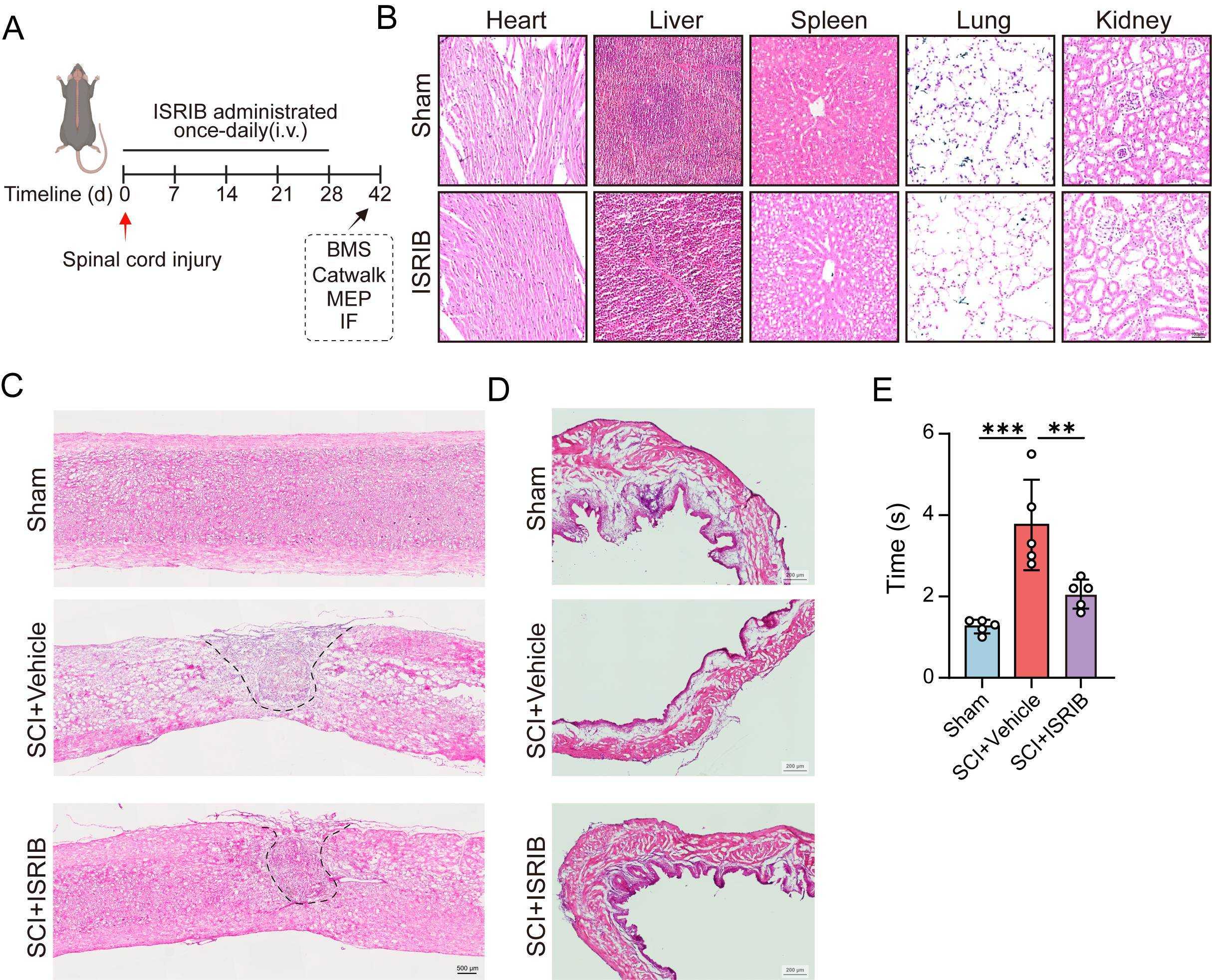


**Fig. S5.** **ISRIB promoted function recovery after spinal cord injury.** (A) Timeline of in vivo experiments in mice. (B) HE staining of heart, liver, spleen, lung, and kidney tissues in Sham group and ISRIB treatment group. Scale bar: 100μm. (C) HE staining of spinal cord tissues. Scale bar: 500μm.(D)Thickness of mice bladder was measured by H&E staining in the Sham, SCI and SCI+ISRIB groups. Scale bar: 200μm. (E)Reaction time to hot plate of hindlimbs in the SCI mice treated with ISRIB (n=5). Data analysis was performed by One-way ANOVA. The data are presented as the means ± SD, **p < 0.01, ***p<0.001.
